# The role of *Helicobacter suis*, *Fusobacterium gastrosuis*, and the pars oesophageal microbiota in gastric ulceration in slaughter pigs receiving meal or pelleted feed

**DOI:** 10.1186/s13567-024-01274-1

**Published:** 2024-02-05

**Authors:** Emily Taillieu, Steff Taelman, Sofie De Bruyckere, Evy Goossens, Ilias Chantziaras, Christophe Van Steenkiste, Peter Yde, Steven Hanssens, Dimitri De Meyer, Wim Van Criekinge, Michiel Stock, Dominiek Maes, Koen Chiers, Freddy Haesebrouck

**Affiliations:** 1 Department of Pathobiology, Pharmacology and Zoological Medicine, Faculty of Veterinary Medicine, Ghent University, Merelbeke, Belgium; 2 Department of Data Analysis and Mathematical Modelling, BIOBIX, Ghent University, 9000 Ghent, Belgium; 3 Department of Data Analysis and Mathematical Modelling, KERMIT, Ghent University, 9000 Ghent, Belgium; 4 BioLizard Nv, Ghent, Belgium; 5 Department of Internal Medicine, Reproduction and Population Medicine, Faculty of Veterinary Medicine, Ghent University, Merelbeke, Belgium; 6 Department of Gastroenterology and Hepatology, University Hospital Antwerp, Antwerp University, Edegem, Belgium; 7 Department of Gastroenterology and Hepatology, General Hospital Maria Middelares, Ghent, Belgium; 8 Danis Nv, Koolskamp, Belgium; 9 Vedanko Bvba, Koolskamp, Belgium

**Keywords:** *Sus scrofa domesticus*, stomach, gastric ulceration, microbiota, *Helicobacter suis*, *Fusobacterium gastrosuis*

## Abstract

This study investigated the role of causative infectious agents in ulceration of the non-glandular part of the porcine stomach (*pars oesophagea*). In total, 150 stomachs from slaughter pigs were included, 75 from pigs that received a meal feed, 75 from pigs that received an equivalent pelleted feed with a smaller particle size. The *pars oesophagea* was macroscopically examined after slaughter. (q)PCR assays for *H. suis*, *F. gastrosuis* and *H. pylori*-like organisms were performed, as well as *16S rRNA* sequencing for *pars oesophagea* microbiome analyses. All 150 pig stomachs showed lesions. *F. gastrosuis* was detected in 115 cases (77%) and *H. suis* in 117 cases (78%), with 92 cases (61%) of co-infection; *H. pylori*-like organisms were detected in one case. Higher infectious loads of *H. suis* increased the odds of severe gastric lesions (OR = 1.14, *p* = 0.038), while the presence of *H. suis* infection in the pyloric gland zone increased the probability of pars oesophageal erosions [16.4% (95% CI 0.6–32.2%)]. The causal effect of *H. suis* was mediated by decreased pars oesophageal microbiome diversity [−1.9% (95% CI − 5.0–1.2%)], increased abundances of *Veillonella* and *Campylobacter* spp., and decreased abundances of *Lactobacillus*, *Escherichia-Shigella*, and *Enterobacteriaceae* spp. Higher infectious loads of *F. gastrosuis* in the *pars oesophagea* decreased the odds of severe gastric lesions (OR = 0.8, *p* = 0.0014). Feed pelleting had no significant impact on the prevalence of severe gastric lesions (OR = 1.72, *p* = 0.28). *H. suis* infections are a risk factor for ulceration of the porcine *pars oesophagea*, probably mediated through alterations in pars oesophageal microbiome diversity and composition.

**Supplementary Information:**

The online version contains supplementary material available at 10.1186/s13567-024-01274-1.

## Introduction

Gastric ulceration is a common health issue in pigs worldwide. Unlike in human patients, where gastric ulceration occurs in the glandular part of the stomach, gastric ulceration in pigs almost exclusively occurs in the non-glandular part, the *pars oesophagea*, of the stomach [1]. The *pars oesophagea* encompasses a small region of stratified squamous epithelium in the porcine stomach at the border with the oesophagus. Under normal conditions, the pH at the *pars oesophagea* and cardiac gland zone (upper compartment) is 5 to 7 [2], while the pH at the fundic and pyloric gland zone (lower compartment) is 2 to 3 and no mixing of luminal content takes place between the two compartments [3]. Since the *pars oesophagea* is not covered by mucus, it is particularly susceptible to irritation and, therefore, also to inflammation. Chronic irritation may occur when the pH barrier between the two compartments disappears, leading to increased contact of the *pars oesophagea* with gastric acid, pepsin and bile salts, and may progress to hyperkeratosis, erosion and, finally, ulceration [3]. Gastric ulceration in pigs has prevalence rates of up to 93%, with the highest prevalence rates in pigs at slaughter age and sows around parturition, and it has been associated with decreased feed intake, decreased daily weight gain and sudden death [1, 4]. It, therefore, significantly impacts animal health, welfare, and production.

The etiology of gastric ulceration of the porcine stomach is multifactorial, with main risk factors being involved in increasing the fluidity of the gastric content. These include small particle size of feed, pelleting of feed and interruption of feed intake [1]. Grain that is ground using a hammer mill may be more ulcerogenic because of a high chance of shattering of the grain kernel, and therefore, using a roller mill is preferred [5]. Also, management strategies, genetic background, hormonal influences, the gastric microbiome and infectious agents have been suggested to be involved, but the exact pathophysiological mechanism is not yet completely understood [3].

*Helicobacter* (*H.*) *suis* is known to colonize the fundic and pyloric gland zone of the porcine stomach, with prevalence rates of up to 60% or more in pigs at slaughter age [3, 6, 7]. It is hypothesized that *H. suis* plays a role in altering the microbiota composition of the *pars oesophagea*, by decreasing the gastric acid production during the acute phase of infection. Alterations in the number and/or function of parietal, somatostatin-producing D- and gastrin-producing G-cells have been observed in naturally *H. suis*-infected 6- to 8-month-old pigs. In these *H. suis*-infected pigs, increased colonization and invasion of the *pars oesophagea* by *Fusobacterium* (*F.*) *gastrosuis* was observed compared to non-infected pigs of the same age, among other shifts in the pars oesophageal microbiome. Epithelial cell death-inducing metabolites produced by *F. gastrosuis* may play a role in gastric ulceration of the porcine stomach. Indeed, preliminary in vitro cell death data point towards a potential role of *F. gastrosuis* in developing porcine gastric ulceration. Furthermore, upregulation of gastric acid production has been observed in the more chronic phase of *H. suis* infection, which may lead to irritation of the *pars oesophagea* [8]. Although the relationship between the presence of *H. suis* in the pyloric and fundic gland zone and the development of gastric ulceration in the porcine stomach has been thoroughly substantiated, the relationship with *F. gastrosuis* remains unclear.

*H. pylori*-like organisms have also been detected in the porcine stomach. These are similar to *H. pylori* isolated from humans, however, distinct from *H. suis*, and may also be associated with gastric ulceration of the porcine stomach [9]. Experimental infection in gnotobiotic piglets with an isolate of these *H. pylori*-like organisms, recovered from a conventionally reared piglet, has resulted in the development of gastritis and gastric ulceration [10]. Recently, Nunes Cortez et al*.* reported the possible presence of *H. pylori*-like organisms in pigs, which were most frequently detected in the *pars oesophagea*, and their significant correlation with erosion [11]. However, these organisms have not yet been fully characterized and their significance remains to be explored.

The objective of this study was to investigate the role of *H. suis*, *F. gastrosuis*, *H. pylori-*like organisms, and changes in the pars oesophageal microbiome in the development and severity of gastric lesions in the *pars oesophagea* in addition to the impact of a finely ground, pelleted feed.

## Materials and methods

### Sampling of the pigs’ stomachs

In total, stomachs of 150 pigs at slaughter age were obtained. The pigs originated from six different barns from the same farm. The genetical and environmental backgrounds were identical for all pigs. On three different dates (25 October 2021, 27 October 2021 and 15 December 2021), each time 25 stomachs of pigs that were fed a less finely ground, meal feed, and 25 stomachs of pigs fed a more finely ground, pelleted feed derived from the meal feed, were obtained. The pigs were fasted for approximately 24 h before slaughter and had always access to drinking water. The stomachs were sampled at the slaughterhouse, at random without any predefined selection. Details concerning the absolute particle sizes of both types of feed based on wet and dry sieving tests are shown in Table 1 and the composition of the feeds in Additional file 1. The stomachs were transported from the slaughterhouse to the laboratory of the faculty of veterinary medicine, Ghent University, and immediately processed. Using scissors that were sterilized using a bead sterilizer, the stomachs were opened along the greater curvature and the insides were rinsed with sterile tap water. Photographs of the *pars oesophagea* of each stomach were taken and the mucosal lesions of the *pars oesophagea* were scored by two independent observers on-site based on the method of Hessing et al. [12]. This is a macroscopic lesion score ranging from 0 to 5, with score 0 for normal mucosa, score 1 for mild hyperkeratosis covering less than 50% of the surface, score 2 for severe hyperkeratosis covering more than 50% of the surface, score 3 for hyperkeratosis with less than five erosions, score 4 for hyperkeratosis with five to ten erosions and score 5 for hyperkeratosis with more than 10 erosions, ulceration or stenosis. From each stomach, a biopsy sample was taken from the *pars oesphagea*, the fundic gland zone and the pyloric gland zone using 8 mm disposable biopsy punches (kai Europe GmbH, Germany). Additionally, another biopsy sample was taken from the *pars oesophagea* of each stomach, which was snap-frozen using liquid nitrogen for microbiome analysis. All biopsy samples were stored at −20 °C until further processing.

**Table 1:** **Wet and dry sieving results determining the absolute particle sizes of the types of feed administered to the slaughter pigs**

Animal weight	Meal feed			Pelleted feed
	Dry sieving		Wet sieving	Wet sieving
	% < 1 mm	GMD	% < 1 mm	% < 1 mm
20–45 kg	68.0	620	77.9	86.4
	71.1	588	77.9	86.3
	69.3	613	77.0	85.4
45–80 kg	67.0	635	78.0	85.6
	69.8	605	80.2	85.7
	69.2	609	79.5	85.5
80–115 kg	67.2	633	78.2	82.7
	70.0	599	78.0	84.7
	68.3	621	77.9	84.2

The more finely ground, pelleted feeds were obtained by grounding the meal type feeds using a hammer mill. GMD = geometric mean diameter.

### PCR and qPCR for the detection and quantification of *Helicobacter suis*

DNA was extracted from the gastric biopsy samples of the *pars oesophagea*, the fundic gland zone and the pyloric gland zone, separately, using the DNeasy Blood & Tissue kit (Qiagen, Hilden, Germany) according to the manufacturer’s instructions.

By means of a *H. suis*-specific qPCR assay based on the *ureAB* gene, the presence and copy number of *H. suis* DNA were determined, according to a previously performed protocol [7]. A standard was included in this assay consisting of tenfold dilutions, starting at 10^8^ PCR amplicons, of a 1236 bp segment of the *ureAB* gene from *H. suis* strain HS5. The obtained copy number was used to calculate the number of *H. suis* bacteria per mg gastric tissue. For the assay, 2 µL of extracted DNA was added to 10 µL reaction mixture, containing 1 × SensiMix™ SYBR No-ROX (Bioline Reagents Ltd, London, UK), 0.5 µM forward primer (BFHsuis_F1) and 0.5 µM reverse primer (BFHsuis_R1), both located within the 1236 bp fragment of the standard. Details on the primer sequences can be found in Table 2. The protocol for qPCR amplification was as follows: initial denaturation for 10 min at 94 °C, 47 cycles of 20 s at 95 °C, 20 s at 62 °C and 30 s at 72 °C, after which the total fluorescence of the samples was measured. Both standards and samples were run in duplicate on a CFX384™ qPCR System with a C1000 Thermal Cycler (Bio-Rad, Hercules, California, USA).

**Table 2:** **Details on qPCR and PCR primers**

Taxon	Target gene	Primer	Primer sequence	PCR product size (bp)	Reference
(q)PCR assays					
*H. suis*	*UreAB*	BFHsuis_F1	FW (5'-AAA ACA MAG GCG ATC GCC CTG TA-3')*	150	[7]
		BFHsuis_R1	RV (5'-TTT CTT CGC CAG GTT CAA AGC G-3')		
*F. gastrosuis*	*gyrB*	GB_2F	FW (5'-GAA GAC AAC CCA GCT GTA ACA-3')	142	[8]
		GB_2R	RV (5'-CAG CTA ATT TCC CAG GAA GTG A-3')		
PCR assay					
*H. pylori*	*UreAB*	BFHpyl_F1	FW (5'-AAA GAG CGT GGT TTT CAT GGC G-3')	217	[13]
		BFHpyl_R1	RV (5'-GGG TTT TAC CGC CAC CGA ATT TAA-3')		
*H. pylori*	*glmM* (*UreC*)	Hpy3F	FW (5'-TTATCGGTAAAGACACCAGAAA-3')	144	[14]
		Hpy3R	RV (5'-ATCACAGCGCATGTCTTC-3')		

^*^M = A or C.

In addition, a *H. suis*-specific PCR assay, using the same primers as in the qPCR assay, was performed (Table 2). The PCR assay was performed in 20 µL reaction volume: 2.5 mM MgCl_2_ (Promega), 1 × GoTaq^®^ Flexi PCR buffer (Promega), 200 µM dNTPs (Bioline), 0.5 µM forward primer, 0.5 µM reverse primer, 0.6 U GoTaq^®^ G2 Flexi DNA polymerase (Promega) and 1 µL of the DNA sample. The protocol for PCR amplification was as follows: pre-incubation for 3 min at 95 °C, 40 cycles of 60 s at 94 °C, 60 s at 59 °C and 60 s at 72 °C, followed by a final completion step for 5 min at 72 °C. As a positive control, genomic DNA of *H. suis* HS5 was used. For visualization and analysis of the PCR assay, 5 µL of each PCR product was analyzed through gel electrophoresis in 1.5% agarose (AGRMP-RO Roche, Merck KGaA, Darmstadt, Germany) with Midori Green (NIPPON Genetics, Düren, Germany) in TBE buffer (VWR Life Science, Amsterdam, The Netherlands). GeneRuler 100 bp Plus DNA Ladder (Thermo Scientific™ SM0323) was used as a weight marker. Images were acquired on a UV transilluminator (UVP PhotoDoc-it Imaging Systems, Fisher Scientific, Hampton, NH, USA).

### PCR and qPCR for the detection and quantification of *Fusobacterium gastrosuis*

The same DNA samples were used as the ones used for *H. suis* PCR and qPCR assays.

A *F. gastrosuis*-specific qPCR based on the *gyrB* gene was performed, to determine the presence and copy number of *F. gastrosuis* DNA, according to a previously performed protocol [8]. A standard was included in this assay consisting of tenfold dilutions, starting at 10^8^ PCR amplicons, of a 1212 bp segment of the *gyrB* gene from *F. gastrosuis* strain CDW1. The obtained copy number was used to calculate the number of *F. gastrosuis* bacteria per mg gastric tissue. For the assay, 2 µL of extracted DNA was added to 10 µL reaction mixture, containing 1 × SensiMix™ SYBR No-ROX (Bioline Reagents Ltd, London, UK), 0.5 µM forward primer (GB_2F) and 0.5 µM reverse primer (GB_2R), both located within the 1212 bp fragment of the standard. Details on the primer sequences can be found in Table 2. The protocol for qPCR amplification was as follows: initial denaturation for 10 min at 95 °C, 47 cycles of 20 s at 95 °C, 30 s at 60 °C and 30 s at 72 °C, after which the total fluorescence of the samples was measured. Both standards and samples were run in duplicate on a CFX384™ qPCR System with a C1000 Thermal Cycler (Bio-Rad, Hercules, California, USA).

A *F. gastrosuis*-specific PCR assay, using the same primers as in the qPCR assay, was also performed (Table 2). The PCR assay was performed in 20 µL reaction volume: 2.5 mM MgCl_2_ (Promega), 1 × GoTaq® Flexi PCR buffer (Promega), 200 µM dNTPs (Bioline), 0.2 µM forward primer, 0.2 µM reverse primer, 0.6 U GoTaq® G2 Flexi DNA polymerase (Promega) and 1 µL of the DNA sample. The protocol for PCR amplification was as follows: pre-incubation for 10 min at 95 °C, 35 cycles of 20 s at 94 °C, 30 s at 60 °C and 30 s at 72 °C, followed by a final completion step for 10 min at 72 °C. For visualization and analysis of the PCR assay, gel electrophoresis was performed as described above.

### PCR for the detection of *Helicobacter pylori*-like organisms

Methods used for the detection of *H. pylori*-like organisms were based on previous work of Cortez Nunes et al*.* [11]. This involved a *H. pylori*-specific PCR assay based on the *ureAB* gene [11, 13], followed by a *H. pylori*-specific PCR assay based on the *glmM* gene [11, 14] which was performed in samples positive in the former PCR assay and allowed to discriminate between *H. pylori* and *H. pylori*-like organisms. Since no reports have been made of pigs naturally infected with *H. pylori*, the latter assay (*glmM*-based) was expected to be negative.

Both PCR assays were performed using the DNA extracted as described above. Each PCR reaction volume consisted of 20 µL containing 2.5 mM MgCl_2_ (Promega), 1 × GoTaq® Flexi PCR buffer (Promega), 200 µM deoxynucleotide triphosphates (dNTPs) (Bioline), forward primer (0.25 µM BFHpyl_F1; 0.5 µM Hpy3F), reverse primer (0.25 µM BFHpyl_R1; 0.5 µM Hpy3R), 0.6 U GoTaq^®^ Flexi DNA polymerase (Promega) and 1 µL of the DNA sample. Details on the primer sequences can be found in Table 2. The protocol for PCR amplification was as follows: pre-incubation for 4 min at 95 °C, 45 cycles of 30 s at 94 °C, 30 s at 59 °C and 1 min at 72 °C, followed by a final completion step for 10 min at 72 °C. For PCR amplification based on the *glmM* gene, the protocol was as follows: pre-incubation for 15 min at 94 °C, 45 cycles of 45 s at 94 °C, 45 s at 58 °C and 45 s at 72 °C, followed by a final completion step for 7 min at 72 °C. As a positive control, genomic DNA of the *H. pylori* strain SS1 was used. For visualization and analysis of the PCR assay, gel electrophoresis was performed as described above.

### Sequencing of positive PCR products

The PCR products of samples with a positive PCR result were sent to Eurofins Genomics^®^ (Edersberg, Germany) for bidirectional Sanger sequencing, in order to avoid false positive results and confirm the identity of the detected species. Sequence editing and assembly of the received amplicon sequences was done using BioNumerics^®^ software (version 7.6.3, Applied Maths, Sint-Martens-Latem, Belgium) and the contig sequences were subjected to the basic local alignment search tool (BLAST) of the NCBI using the non-redundant nucleotide database [15]. A cut-off value of 96% was used for average nucleotide identity as a threshold for species delineation [16].

### Statistical analysis of qPCR and feed type data

Since the data concerning infectious loads of *H. suis* and *F. gastrosuis* were not normally distributed, the natural logarithms of these data were calculated for further analyses. The presence and abundance of *H. suis* and *F. gastrosuis* in the sampled regions of the stomach together with the types of feed administered were included as fixed effects in a binomial mixed effects logistic regression model, to determine the independent risks associated with each of these factors for the severity of gastric lesions in the *pars oesophagea*. In addition, ordinal logistic regression was performed to investigate the independent contributions of the presence and abundance of *H. suis* and *F. gastrosuis* in the sampled stomach regions to the severity of gastric lesions in the *pars oesophagea*. Wilcoxon Signed Rank tests with Bonferroni correction, to counteract the multiple comparisons problem, were performed for comparison of infectious loads between groups of pigs with different macroscopic lesion scores. In another binomial mixed effects logistic regression model, the independent contribution of a finely ground, pelleted feed to the presence of *H. suis* was determined. The barns which the pigs originated from were taken into account as a random variable where needed. To obtain final logistic regression models, forward stepwise addition of independent variables which showed no interaction with other variables in the model was performed. This type of final logistic regression was performed to determine the variables with the highest predictive power. Odds ratios with their corresponding 95% confidence intervals and *p*-values were calculated for potential risk factors. A two-sided *p*-value of ≤ 0.05 was considered statistically significant. Statistical analyses were performed using R version 4.0.3.

### *16S rRNA* sequencing for microbiome analyses

DNA was extracted from snap-frozen pars oesophageal samples using the QIAamp PowerFecal® Pro DNA Kit (Qiagen, Hilden, Germany) according to the manufacturer’s instructions since this kit yielded robust results for *16S rRNA* sequencing. The *16S rRNA* V3-V4 hypervariable region was amplified using the following primers: S-d-Bact-0341-b-S-17 (5′-TCGTCG GCA GCG TCA GAT GTG TAT AAG AGA CAG CCTACGGGNGGC WGC AG-3′) and S-d-Bact-0785-a-A-21 (5′-GTC TCG TGG GCT CGG AGA TGT GTA TAA GAGACA GGA CTACHVGGG TAT CTA ATC C-3′) [17]. The PCR assay was performed in 25 µL reaction volume: 1 × KAPA HiFi HotStart ReadyMix (Roche, Diegem, Belgium), 0.2 µM forward primer, 0.2 µM reverse primer and 2.5 µL of the DNA sample. The protocol for PCR amplification was as follows: pre-incubation for 3 min at 95 °C, 25 cycles of 30 s at 95 °C, 30 s at 55 °C and 30 s at 72 °C, followed by a final completion step for 5 min at 72 °C. Purification of the PCR products was achieved using CleanNGS beads (CleanNA, Waddinxveen, The Netherlands) and the DNA quantity and quality was analyzed through gel electrophoresis as described above. An index PCR was performed to attach dual indices and Illumina sequencing adapters (i5 and i7 primers) to the obtained amplicons. The PCR assay was performed in 50 µL reaction volume: 1 × KAPA HiFi HotStart ReadyMix (Roche, Diegem, Belgium), 0.5 µM forward primer, 0.5 µM reverse primer and 5 µL of the purified PCR product. The protocol for PCR amplification was as follows: pre-incubation for 3 min at 95 °C, 8 cycles of 30 s at 95 °C, 30 s at 55 °C and 30 s at 72 °C, followed by a final completion step for 5 min at 72 °C. Again, purification of the obtained PCR products was achieved and the DNA quantity and quality was analyzed through gel electrophoresis as described above. The DNA concentration was determined using the Quantus fluorimeter (Promega, Leiden, Netherlands). The final barcoded libraries were combined to an equimolar 5 nM pool and sequenced using Illumina MiSeq v3 technology (2 × 300 bp, paired-end) at Macrogen (Amsterdam, The Netherlands). Adapter sequences were trimmed from the reads using TrimGalore v0.6.10 [18] and quality was monitored using multiQC v1.14 [19]. Paired-end reads were assembled into Amplicon Sequence Variants (ASVs) using DADA2 v1.18.0 [20] and taxonomically annotated using DECIPHER v2.18.1 and the SILVA r138.1 reference dataset [21, 22]. Likely contaminants found in the PCR control, DNA extraction control, and environmental contaminant controls were removed using decontam v1.10.0 [23]. Specific ASVs of interest that could not be taxonomically annotated with certainty by DECIPHER and the SILVA database, were run through BLASTn with the 16S ribosomal RNA database [24].

### Causal statistics of *16S rRNA* amplicon data—mediation and differential abundance analyses

**Figure 1:**
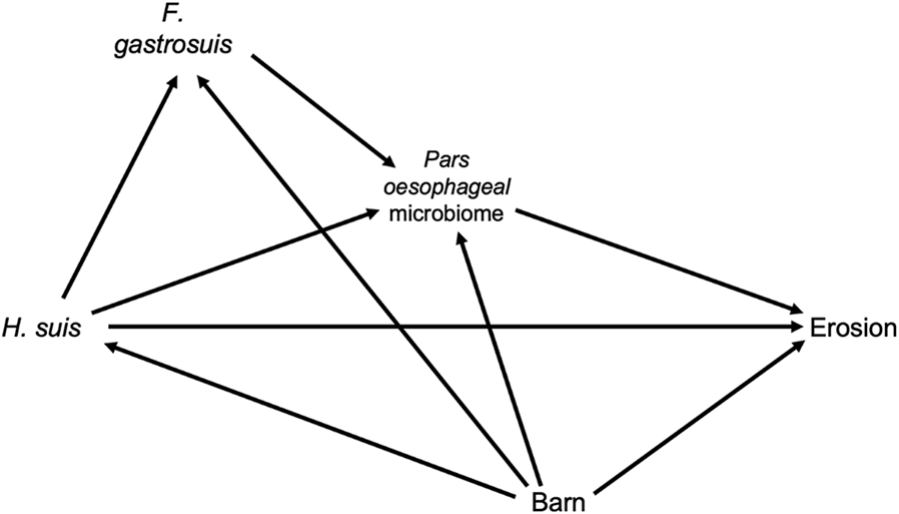
**Causal Directed Acyclic Graph (DAG) of the assumptions made for the causal mediation analyses.** *H. suis* denotes the presence of the bacterium in the fundic and pyloric gland zones, based on the qPCR assay. *F. gastrosuis* denotes the presence of the bacterium in the *pars oesophagea* based on the qPCR assay. The pars oesophageal microbiome is quantified by either its Shannon diversity index or the Amplicon Sequence Variant (ASV) counts of individual taxa. Due to their collinearity, the feed type is fully captured in the barn variable. The presence of erosion was defined as a macroscopic lesion score higher than two.

The causal Directed Acyclic Graph (DAG) in Figure 1 sets out the assumptions made for all causal analyses. For consistency, the exposures were defined as the binarized presence of *H. suis* in the fundic and pyloric gland zone, and the outcome was defined as the presence of erosion (a macroscopic lesion score higher than 2). The pars oesophageal microbiome was defined as a mediator between exposure and outcome, and it was quantified by either its Shannon diversity index as calculated in bits using phyloseq v1.44.0 [25] or by the individual Amplicon Sequence Variants (ASV) counts. Shannon diversity indices were translated to effective numbers of species using the formulas posed by Jost [26]. A sensitivity analysis of the impact of any unobserved confounders was conducted between the presence of *H. suis* and the Shannon diversity index using sensemakr v0.1.4 [27]. Mediation analysis was conducted using medoutcon v0.2.0 with binomial g-, h-, and b-learners from sl3 v1.4.5 [28]. Differential Abundance Analyses (DAA) were conducted to assess the association between (i) microbial abundances and feed type and (ii) microbial abundances and macroscopic lesion score. These were executed using treeclimbr v0.1.5 and edgeR v3.42.4 [29]. Based on the causal DAG, the DAA on feed type was unadjusted, while the analysis on lesion score was adjusted for both barn and *H. suis* presence in the fundic and pyloric gland zone. The *p*-value threshold to assure a False Discovery Rate (FDR) of 0.05 was calculated using permFDP v0.1.0 [30].

## Results

### Macroscopic lesion scoring of the *pars oesophagea*

**Figure 2:**
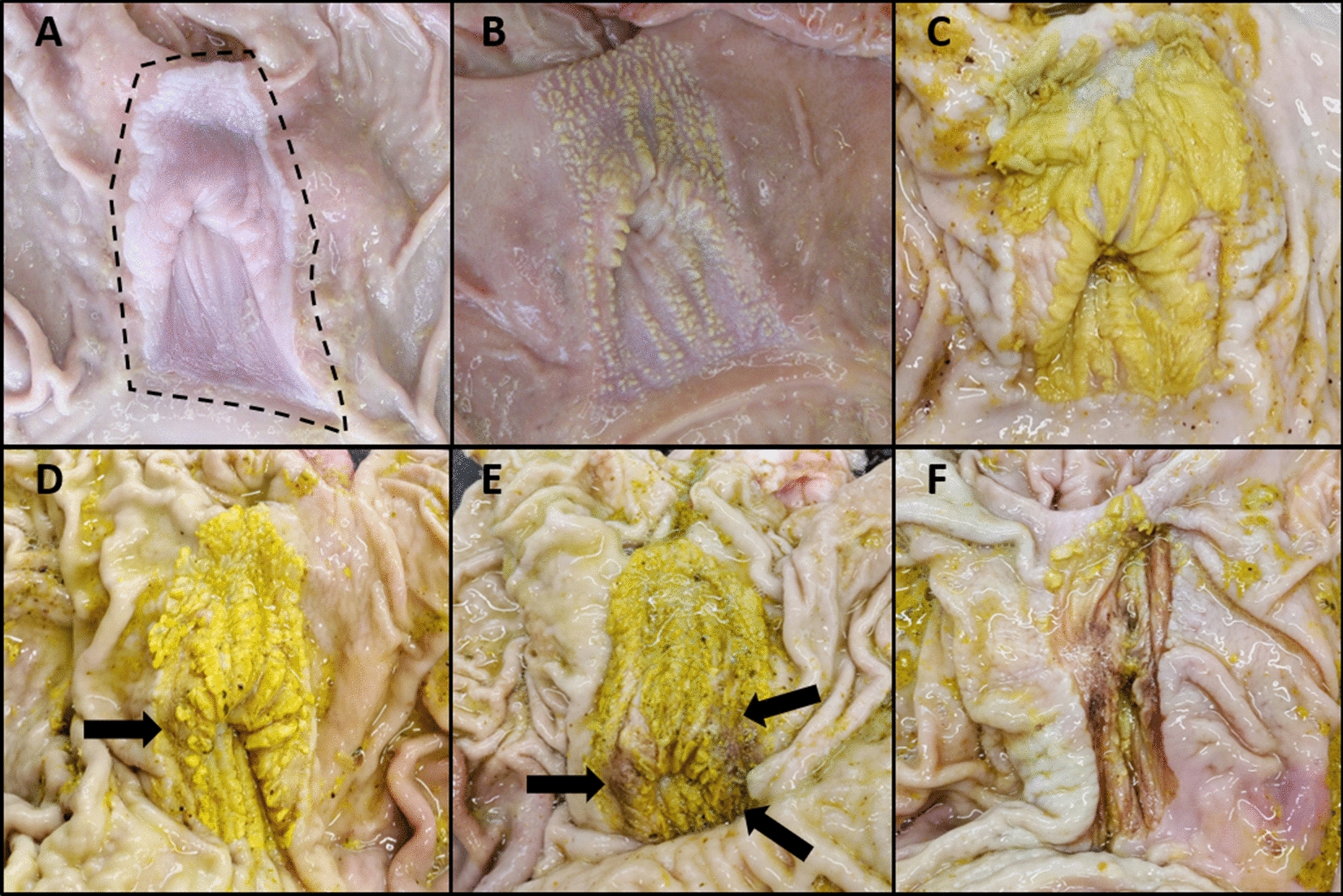
**Lesions in the *pars oesophagea* of slaughter pigs according to the method of Hessing et al*.*** [12]. **A** Score 0 = normal mucosa (the *pars oesophagea* is delineated by a black dotted line and appears smooth and glistening), **B** Score 1 = mild hyperkeratosis covering less than 50% of the surface, **C** Score 2 = severe hyperkeratosis covering more than 50% of the surface, **D** Score 3 = hyperkeratosis with few erosions (erosions are indicated by black arrows), **E** Score 4 = hyperkeratosis with several erosions (erosions are indicated by black arrows), **F** Score 5 = ulceration. The yellowish staining of affected, rough and thickened *pars oesophagea* mucosa is the result of bile staining [1]. Pictures 1A and 1B are from an earlier, unpublished study since no stomachs with scores 0 or 1 were observed in the current study.

No pigs showed a normal mucosa of the *pars oesophagea* or showed a mucosa with mild hyperkeratosis. Hence, all pig stomachs received a score ranging from 2 to 5. The distribution of the macroscopic lesion scores among all examined stomachs, per feed type administered, is presented in Table 3. Overall, pigs fed a more finely ground, pelleted feed showed severe lesions (score 4 or 5) more often (31/75 or 41.3%) compared to pigs fed a less finely ground, meal feed (22/75 or 29.3%). Figure 2 gives an overview of the different macroscopic lesions in the *pars oesophagea* observed in the stomachs of the slaughter pigs examined in this study.

**Table 3:** **Overview of the score distribution of lesions in the *pars oesophagea *of slaughter pigs according to feed type**

Type of feed administered	Macroscopic lesion score					
	0 (n (%))	1 (n (%))	2 (n (%))	3 (n (%))	4 (n (%))	5 (n (%))
Less finely ground, meal feed (N = 75)	0 (0%)	0 (0%)	26 (34.7%)	27 (36%)	15 (20%)	7 (9.3%)
Finely ground, pelleted feed (N = 75)	0 (0%)	0 (0%)	24 (32%)	20 (26.7%)	23 (30.7%)	8 (10.7%)
Total (N = 150)	0 (0%)	0 (0%)	50 (33.3%)	47 (31.3%)	38 (25.3%)	15 (10%)

0 = normal mucosa, 1 = mild hyperkeratosis covering less than 50% of the surface, 2 = severe hyperkeratosis covering more than 50% of the surface, 3 = hyperkeratosis with few erosions, 4 = hyperkeratosis with several erosions and 5 = hyperkeratosis with many erosions, ulceration or stenosis.

### Presence and quantification of *Helicobacter suis* and *Fusobacterium gastrosuis* and presence of *Helicobacter pylori*-like organisms

Using qPCR methods, *H. suis* was detected in the pyloric and/or fundic gland zone of 117 stomachs (78%) and *F. gastrosuis* in the *pars oesophagea* of 115 stomachs (76.7%). Co-infection with both infectious agents was detected in 92 stomachs (61.3%). PCR and sequencing results confirmed the presence of *H. suis* in 88 out of 92 (95.7%) and 76 out of 89 (85.4%) of fundic gland zone and pyloric gland zone samples positive in qPCR, respectively. For the presence of *F. gastrosuis* in *pars oesophagea* samples, positive qPCR results could be confirmed by PCR and sequencing analysis in 110 out of 115 (95.7%) samples. *H. suis* was also detected in the *pars oesophagea* (qPCR results: 61/150 (40.7%) positive, confirmed by PCR and sequencing analysis in 27 samples), however, at very low infectious loads (16.1 ± 13.75 bacteria/mg tissue). The same accounts for *F. gastrosuis* in the fundic (qPCR results: 9/150 (6%) positive, all confirmed by PCR and sequencing analysis; infectious load = 96.93 ± 163.71 bacteria/mg tissue) and pyloric (qPCR results: 12/150 (8%) positive, confirmed by PCR in 10 samples; infectious load = 34.94 ± 32.98 bacteria/mg tissue) gland zone. In general, both infectious agents were more frequently and more abundantly present in the stomachs of pigs fed a more finely ground, pelleted feed as opposed to the less finely ground, meal feed. All numbers regarding the presence and infectious loads of *H. suis* and *F. gastrosuis* are summarized in Table 4.

**Table 4:** **Presence and abundance of *Helicobacter suis*** **and** ***Fusobacterium gastrosuis* according to qPCR results**

Type of feed administered	Presence of *H. suis* (n (%))	Abundance of *H. suis* (# bacteria/mg tissue)	Presence of *F. gastrosuis* (n (%))	Abundance of *F. gastrosuis* (# bacteria/mg tissue)	Co-presence of *H. suis* and *F. gastrosuis* (n (%))
Less finely ground, meal feed (N = 75)	53 (70.7%)	Fundic gland zone: 112.65 ± 252.81 Pyloric gland zone: 325.33 ± 668.52	55 (73.3%)	192.35 ± 395.11	40 (53.3%)
Finely ground, pelleted feed (N = 75)	64 (85.3%)	Fundic gland zone: 150.92 ± 218.02 Pyloric gland zone: 259.54 ± 496.92	60 (80%)	697.37 ± 3272.88	52 (69.3%)
Total (N = 150)	117 (78%)	Fundic gland zone: 133.86 ± 233.62 Pyloric gland zone: 287.63 ± 573.83	115 (76.7%)	455.84 ± 2383.68	92 (61.3%)

The stomach was considered positive for *H. suis* in case it was detected in either the pyloric or fundic gland zone.

One out of the 150 (0.7%) fundic gland zone samples was positive in the *ureAB*-based *H. pylori*-specific PCR assay, which was confirmed by sequencing, and negative in the *glmM*-based *H. pylori*-specific PCR assay. None of the samples of the *pars oesophagea* and pyloric gland zone of the 150 slaughter pigs examined for the presence of the *ureAB* gene of *H. pylori*-like organisms was positive in PCR analysis.

### Risk factor analyses based on qPCR and feed type data

In Table 5, the models performed to assess potential, independent risk factors for the presence of severe gastric lesions in the *pars oesophagea* of slaughter pigs, are presented together with the results. Since the descriptive macroscopic lesion scoring results showed that pigs fed a more finely ground, pelleted feed showed severe lesions (score 4 or 5) more often compared to pigs fed a less finely ground, meal feed, the outcome was defined as severe gastric lesions (score 4 or 5) versus mild gastric lesions (score 2 or 3), and the main potential risk factor assessed was the finely ground, pelleted feed, as opposed to the less finely ground, meal feed. When performing a simple mixed effects logistic regression model, the less finely ground, pelleted feed did not seem to significantly impact the odds of severe gastric lesions, however, there was a positive trend towards an effect (odds ratio (OR) (95% confidence interval (CI)) = 1.72 (0.64–4.63), *p* = 0.28). When adding the presence and abundance of *H. suis* and *F. gastrosuis* as predictor variables to the simple model, the infectious loads of *H. suis* in the fundic gland zone and of *F. gastrosuis* in the *pars oesophagea* were found to significantly impact the odds of developing severe gastric lesions (1.23 (1.03–1.46), *p* = 0.022 and 0.81 (0.68–0.95), *p* = 0.0087), respectively). In case the infectious load of *H. suis* in the fundic gland zone increased with one ln unit, the odds to develop severe gastric lesions increased by 23% (holding all other variables in the model constant), while the odds decreased by 19% in case the infectious load of *F. gastrosuis* in the *pars oesophagea* increased with one ln unit (holding all other variables in the model constant).

**Table 5:** **Logistic regression analyses to determine risk factors for the presence of severe gastric lesions in the *pars oesophagea***

	Potential risk factor	OR (95% CI)	*p*-value
Simple mixed effects logistic regression model	Finely ground, pelleted feed (vs. meal feed) (Barn included as a random variable with *n* = 6)	1.72 (0.64–4.63)	0.28
Final mixed effects logistic regression model	Finely ground, pelleted feed (vs. meal feed) Infectious load of *H. suis* in fundic gland zone (ln #/mg gastric tissue) Infectious load of *F. gastrosuis* (ln #/mg gastric tissue) (Barn included as a random variable with *n* = 6)	1.71 (0.57–5.13) **1.23 (1.03–1.46)** **0.81 (0.68–0.95)**	0.34 **0.022** **0.0087**

OR: odds ratio, CI: confidence interval.

**Figure 3:**
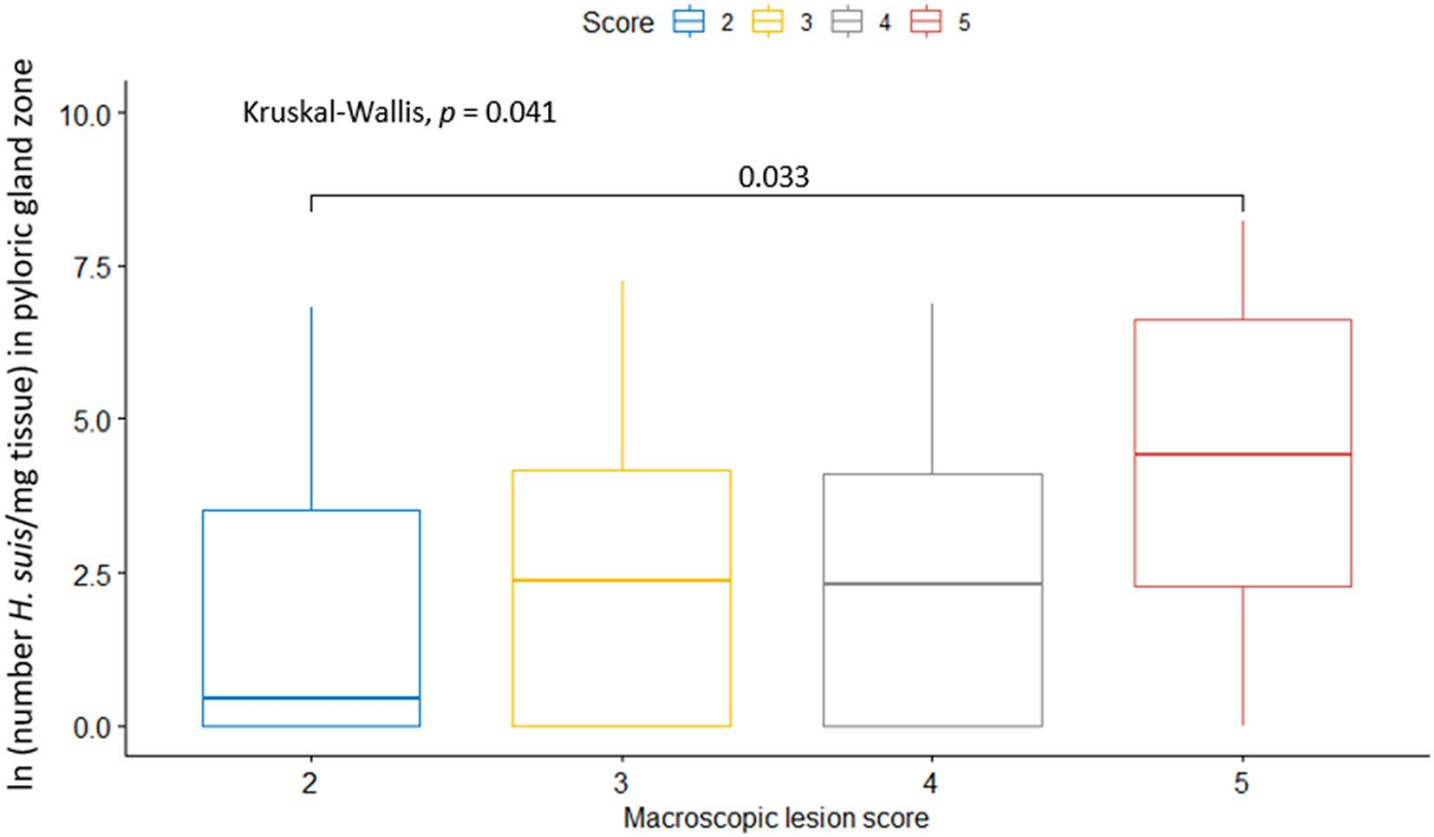
**Comparison of the infectious load of *H. suis* in the pyloric gland zone (in ln scale) between the different macroscopic lesion score groups.** The data are presented as boxplots using standard Tukey representation and are expressed as the natural logarithm of the number of *H. suis* per mg tissue. The statistical comparison between the gastric lesion groups was performed using the non-parametric Kruskal–Wallis H test with Bonferroni correction, which counteracts the multiple comparisons problem.

**Figure 4:**
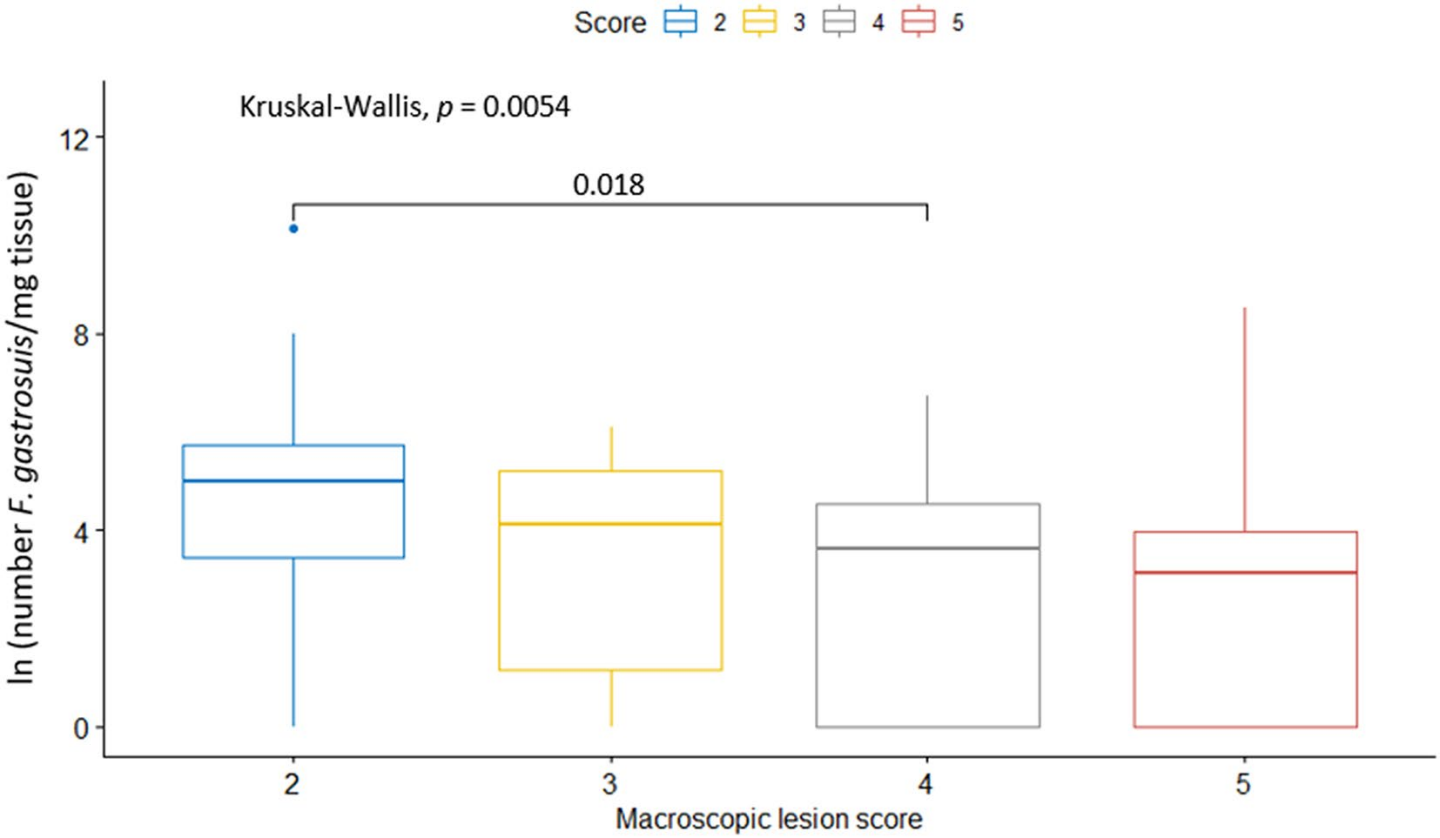
**Comparison of the infectious load of *F. gastrosuis* in the *pars oesophagea* (in ln scale) between the different macroscopic lesion score groups.** The data are presented as boxplots using standard Tukey representation and are expressed as the natural logarithm of the number of *F. gastrosuis* per mg tissue. The statistical comparison between the gastric lesion groups was performed using the non-parametric Kruskal–Wallis H test with Bonferroni correction, which counteracts the multiple comparisons problem.

Logistic regression models were also performed to determine independent risk factors associated with a lower or higher susceptibility to a certain macroscopic lesion score (Table 6). The potential risk factors included the variables regarding the presence and abundance of *H. suis* and *F. gastrosuis*. Simple logistic regression models predicted higher infectious loads of *H. suis* in the fundic and pyloric gland zone to be independent risk factors for a higher macroscopic lesion score (1.16 (1.02–1.32), *p* = 0.027 and 1.15 (1.03–1.3), *p* = 0.020, respectively), while a higher infectious load of *F. gastrosuis* in the *pars oesophagea* was an independent predictor for a lower macroscopic lesion score (0.79 (0.69–0.9), *p* = 0.00078). In the final multiple logistic regression model, the infectious load of *H. suis* in the pyloric gland zone was found to be an independent positive predictor for a higher lesion score, where the odds for the score to increase with one unit was 14% when the infectious load increased with one ln unit (holding all other variables in the model constant). Also in this model, the infectious load of *F. gastrosuis* in the *pars oesophagea* was found to be an independent negative predictor for a higher lesion score, where the odds for the score to decrease with one unit was 20% when the infectious load increased with one ln unit (holding all other variables in the model constant). Additionally, it was checked between which groups of macroscopic lesion scores the greatest difference in infectious loads of *H. suis* in the pyloric gland zone and *F. gastrosuis* in the *pars oesophagea* occurred. For *H. suis*, a statistically significant difference in infectious load was noted only between the groups of pig stomachs that scored 2 and 5 (adjusted *p* = 0.033) (Figure 3). For *F. gastrosuis*, there was a statistically significant difference in infectious load between the groups of pig stomachs that scored 2 and 4 (adjusted *p* = 0.018) and a non-significant difference between the groups of pig stomachs that scored 2 and 5 (adjusted *p* = 0.066) (Figure 4).

**Table 6:** **Logistic regression analyses to determine risk factors for the severity of gastric lesions in the *pars oesophagea***

	Potential risk factor	OR (95% CI)	*p*-value
Simple logistic regression model	Presence of *H. suis* in the fundic or pyloric gland zone (vs. no presence of *H. suis*)	1.03 (0.52–2.08)	0.92
Simple logistic regression model	Infectious load of *H. suis* in fundic gland zone (ln #/mg gastric tissue)	**1.16 (1.02–1.32)**	**0.027**
Simple logistic regression model	Infectious load of *H. suis* in pyloric gland zone (ln #/mg gastric tissue)	**1.15 (1.03–1.3)**	**0.020**
Simple logistic regression model	Infectious load of *F. gastrosuis* (ln #/mg gastric tissue)	**0.79 (0.69–0.9)**	**0.00078**
Final logistic regression model	Infectious load of *H. suis* in pyloric gland zone (ln #/mg gastric tissue) Infectious load of *F. gastrosuis* (ln #/mg gastric tissue)	**1.14 (1.01–1.28)** **0.8 (0.7–0.91)**	**0.038** **0.0014**

OR: odds ratio, CI: confidence interval.

Finally, logistic regression analysis was performed to determine the impact of the finely ground, pelleted feed on the presence of *H. suis* in the fundic and/or pyloric gland zone (Table 7). Although there may be a trend towards an increased odds of being infected with *H. suis* when fed the pelleted feed (2.43 (0.97–6.08)), this result was non-significant (*p* = 0.058).

**Table 7:** **Logistic regression analyses to determine risk factors for the presence of** ***H. suis*** **in the pyloric or fundic gland zone**

	Potential risk factor	OR (95% CI)	***p*-value**
Simple mixed effects logistic regression model = Final model	Finely ground, pelleted feed (vs. meal feed) (Barn as a random variable with *n* = 6)	2.43 (0.97–6.08)	0.058

OR = odds ratio, CI = confidence interval.

### Pars oesophageal microbiome diversity and composition

**Figure 5:**
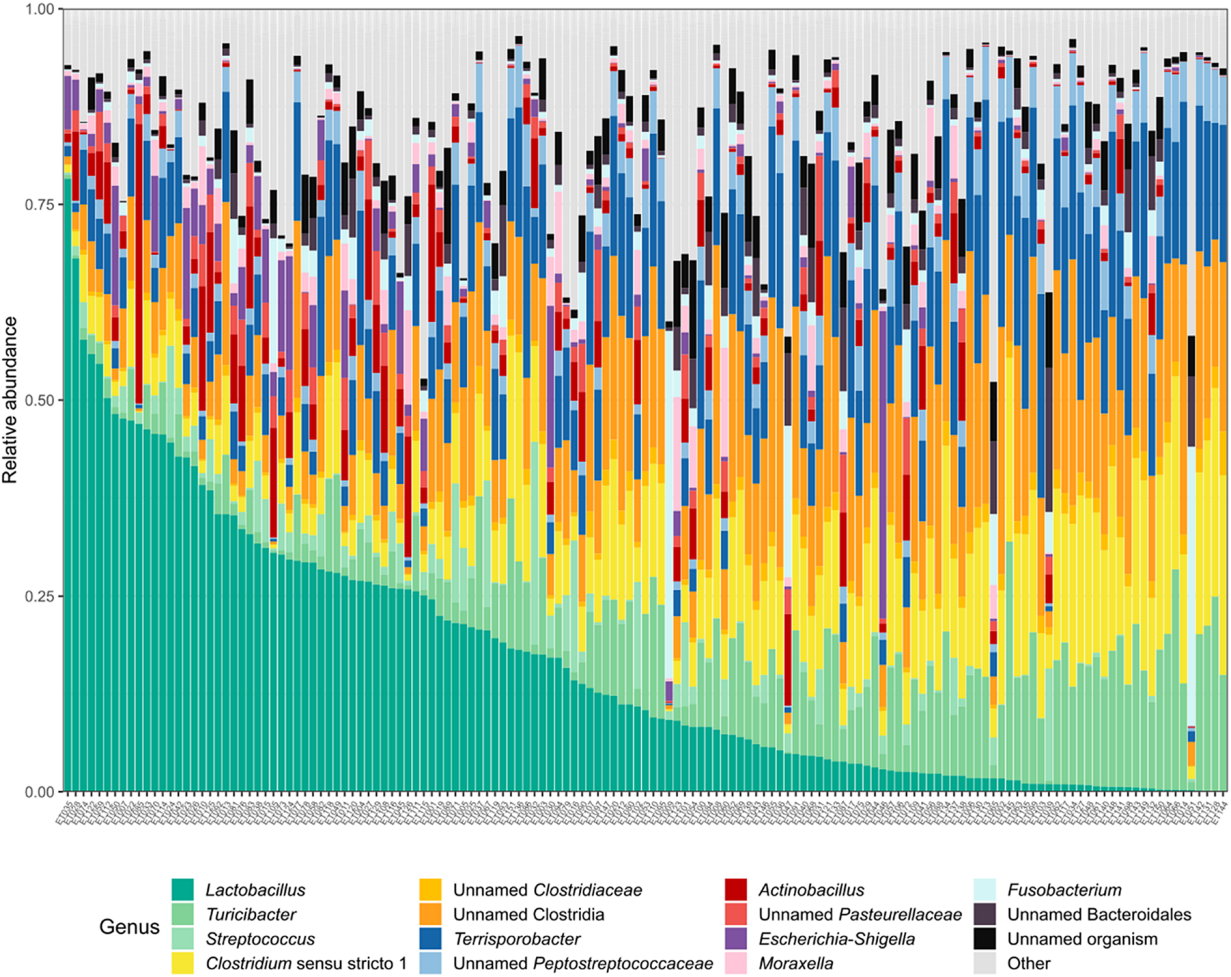
**Relative abundance of the 15 most abundant bacterial genera in each sample from the *pars oesophagea*. **Lesser prominent genera were aggregated into the “Other” group.

In total, 325 unique microbial genera could be annotated to the pars oesophageal sequencing data, with *Lactobacillus*, *Clostridium*, *Terrisporobacter*, and *Turicibacter* as the most dominant genera (Figure 5).

**Figure 6:**
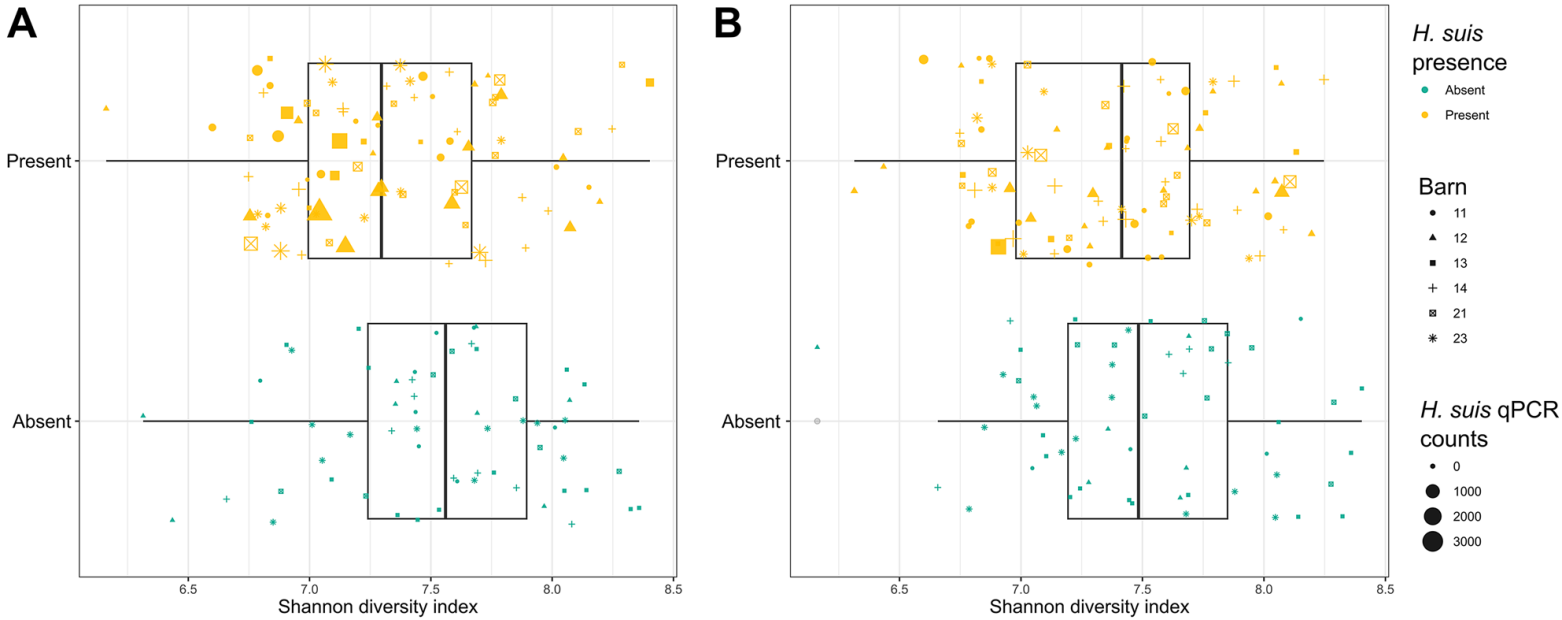
**Boxplots showing the microbial diversity of the *pars oesophagea* in samples where *H. suis* is or is not present in (A) the pyloric or (B) the fundic gland zone**. For each gland zone studied, there is on-average a higher pars oesophageal microbial diversity in samples where *H. suis* is not present.

The within-sample diversity, as expressed by the Shannon diversity index, was shown to be significantly higher when *H. suis* was not present in the pyloric gland zone (−0.18040, (−0.3 to −0.02), *p* = 0.0258), and non-significantly higher when *H. suis* was not present in the fundic gland zone (−0.15033, (−0.32–0.01), *p* = 0.0764). These estimated decreases translate to 1.20 and 1.16 evenly distributed species less when *H. suis* is present in the pyloric or fundic gland zone, respectively (Figure 6).

DAA of the microbial abundances against feed type revealed higher abundances of *Lactobacillus* and *Terrisporobacter* species, and lower abundances of *Clostridium* sensu stricto 1 species in the *pars oesophagea* of animals that received meal feed rather than pelleted feed (Table 8). Of these, only the *Lactobacillus* species’ abundance showed a difference with log_2_ Fold Change (logFC) higher than 1.

**Table 8:** **Top 10 differentially abundant Amplicon Sequence Variants (ASVs) in the** ***pars oesophagea*** **associated with meal feed.** ***p*-values are unadjusted, but the threshold for 5% False Discovery Rate (FDR) was estimated at 0.013**

ASV taxonomy	logFC	logCPM	*p*-value
s__*Clostridium* sensu stricto 1	−0.46	8.23	0.000139
s__*Lactobacillus*	1.43	11.03	0.000425
s__*Clostridium* sensu stricto 1	−0.41	8.06	0.000601
s__*Terrisporobacter*	0.31	9.57	0.000760
s__*Terrisporobacter*	0.27	10.09	0.000929
s__*Clostridium* sensu stricto 1	−0.41	8.15	0.00101
s__*Lactobacillus*	1.36	11.26	0.00103
s__*Lactobacillus*	1.32	11.07	0.00104
s__*Lactobacillus*	1.32	11.90	0.00113
s__*Lactobacillus*	1.36	10.97	0.00115

Full table is available in Additional file 2.

### Pars oesophageal microbiome as a mediator in *Helicobacter suis*-caused gastric erosion

**Figure 7:**
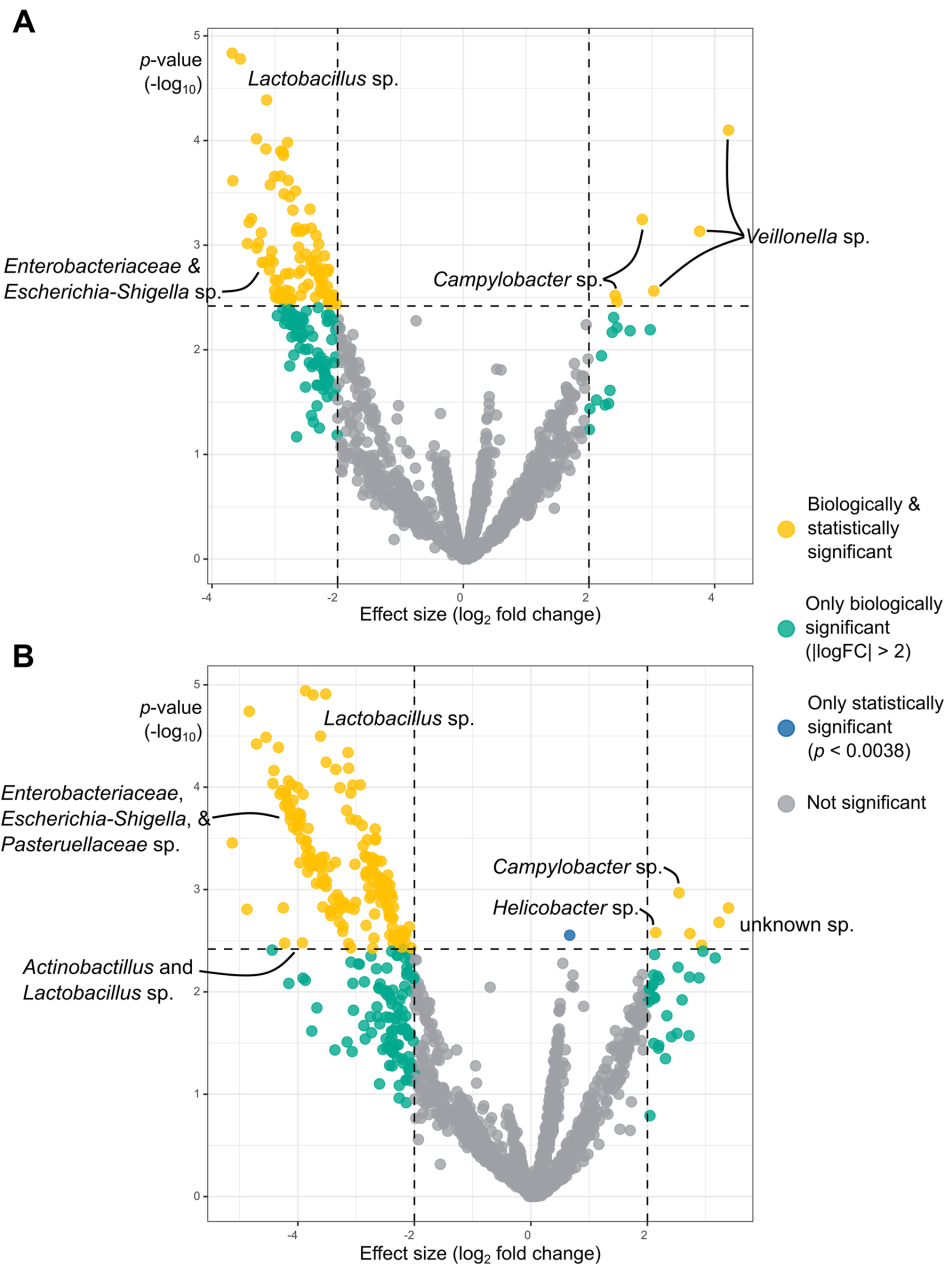
**Volcano plots showing the effect of micro-organisms in the *pars oesophagea* on the macroscopic lesion score.** Given their confounding effect, these analyses are adjusted for barn and presence of *H. suis* in the pyloric gland zone (**A**), or in the fundic gland zone (**B**). Green dots represent taxa of biological significance (high effect sizes between different lesion scores), but no statistical significance. Blue dots represent taxa of only statistical significance [*p*-values lower than 0.0038—the estimated threshold for 5% False Discovery Rate (FDR)]. Yellow dots represent taxa of both biological and statistical significance. Grey dots represent non-significant taxa.

To nullify any observed effect of *H. suis* in the fundic and pyloric gland zones on the pars oesophageal diversity, sensitivity analysis indicated that the effects of any unobserved confounders should be more than eight and more than 45 times the magnitude of the effect of the barns on the pars oesophageal diversity, respectively (Additional file 3). Disregarding any effect through changes in the microbiome, the Natural Direct Effect (NDE) of the presence of *H. suis* in the pyloric gland zone is estimated to increase the probability of erosion by 16.4% (95% CI 0.6–32.2%). The Natural Indirect Effect (NIE), i.e., the effect of the presence of *H. suis* only through changes in the microbiome, is estimated to decrease the probability of erosion by 1.9% (95% CI −5.0–1.2%). For the presence of *H. suis* in the fundic gland zone, the NDE and NIE are respectively estimated to be 7.4% (−10.6–25.3%), and 2.3% (−1.5–6.2%). As can be seen in Figure 7, DAA between the microbiota of the *pars oesophagea* and macroscopic lesion scores reveals that specifically ASVs from *Campylobacter*, *Veillonella*, and *Helicobacter* are statistically and biologically significantly more abundant in case of higher lesion scores. Four significantly more abundant ASVs that could not be taxonomically annotated by DECIPHER were also found. One of these matched a partial sequence of the *16S rRNA* gene from *Actinobacillus minor* with 99% identity. The other three matched with 89% identity to *Alloprevotella rava*. Various *Lactobacillus*, *Actinobacillus*, *Escherichia-Shigella*, and other *Enterobacteriaceae* species were significantly more abundant in case of lesion scores where no erosion was present (Tables 9 and 10).

**Table 9:** **Top 10 differentially abundant Amplicon Sequence Variants (ASVs) in the** ***pars oesophagea*** **associated with high macroscopic lesion score in a Differential Abundance Analysis (DAA) adjusted for the barns and presence of** ***H. suis*** **in the pyloric gland zone**

ASV taxonomy	logFC	logCPM	*p*-value
s__*Veillonella*	4.22	7.18	7.96E-05
s__*Veillonella*	3.76	6.99	0.000736
s__*Veillonella*	3.03	7.06	0.00274
s__*Campylobacter*	2.85	7.29	0.000569
s__*Campylobacter*	2.45	7.02	0.00345
s__*Campylobacter*	2.42	7.31	0.00303
s__*Lactobacillus*	−2.07	8.83	0.00299
s__*Lactobacillus*	−2.07	7.88	0.00349
s__*Lactobacillus*	−2.10	8.089	0.00319
s__*Lactobacillus*	−2.11	7.87	0.00312

*p*-values are unadjusted, but the threshold for 5% False Discovery Rate (FDR) was estimated at 0.0036. Full table is available in Additional file 2.

**Table 10:** **Top 10 differentially abundant Amplicon Sequence Variants (ASVs) in the** ***pars oesophagea*** **associated with high macroscopic lesion score in a Differential Abundance Analysis (DAA) adjusted for the barns and presence of** ***H. suis*** **in the fundic gland zone**

ASV taxonomy	logFC	logCPM	*p*-value
Unnamed_organism_85	3.39	6.80	0.00151
Unnamed_organism_60	3.23	6.90	0.00209
Unnamed_organism_76	2.93	6.80	0.00351
Unnamed_organism_73	2.73	6.82	0.00270
Unnamed_*Campylobacter*_3	2.54	7.22	0.00108
Unnamed_*Helicobacter*_30	2.14	7.21	0.00263
Unnamed_*Clostridium* sensu stricto 1_60	0.67	9.37	0.00280
Unnamed_*Lactobacillus*_86	−2.09	8.78	0.00231
Unnamed_*Lactobacillus*_126	−2.15	8.52	0.00274
Unnamed_*Lactobacillus*_183	−2.16	8.21	0.00329

*p*-values are unadjusted, but the threshold for 5% False Discovery Rate (FDR) was estimated at 0.0036. Full table is available in Additional file 2.

Causal mediation analysis with the significantly more abundant taxa as mediators estimates an indirect effect of *H. suis* in the pyloric gland zone on the probability of erosion of −0.6% (95% CI −3.5–2.3%). The NIE of *H. suis* in the fundic gland zone is estimated at 0.3% (95% CI −3.5–4.0%). The NIEs of the significantly less abundant taxa as mediators in the effect of *H. suis* in the pyloric and fundic gland zone are estimated at −4.2% (95% CI −7.1 to −1.3%) and 1.5% (95% CI −1.4–4.4%), respectively.

## Discussion

The prevalence of gastric lesions in the *pars oesophagea* of slaughter pigs was 100% in this study. This exceeds the already high estimated prevalence of up to 90% [1]. Although all pigs included in the study were reared at the same farm, the stomachs were selected at random, without any preselection for increased susceptibility for gastric lesions, therefore suggesting that gastric ulceration in the *pars oesophagea* is a common health issue in this herd. Possibly, mild lesions may have developed due to the 24-h fasting period prior to slaughter.

Pelleting of the feed, and therefore a smaller particle size of the feed, did not seem to significantly impact the severity of the gastric lesions. This might be explained by the fact that there was no major difference in particle size between the meal and pelleted feed used in this study (on average between 5.8% and 8.4% difference according to animal weight). However, there was a trend towards a positive effect, suggesting that it might play a role in the multifactorial origin of gastric ulceration in pigs as described many times before. Both the absolute smaller particle size of the feed and the production process using a hammer mill to create the pelleted feed might add to this effect [5, 31–33].

The results of the current study confirm that *H. suis* plays a role in the development of gastric lesions in the *pars oesophagea*. The severity of the lesions was significantly impacted by the infectious load of *H. suis*, with a higher infectious load being associated with more severe lesions, rather than the mere occurrence of a *H. suis* infection. To the best of our knowledge, this is the first time that this specific association has been described, while earlier studies reported a positive association between the occurrence of *H. suis* infection and gastric ulceration [7]. Causal analysis, on the other hand, showed that the presence of *H. suis*, mainly in the pyloric gland zone, significantly increased the odds of erosions or ulceration. This effect was found to be mediated by a decrease in pars oesophageal microbiome diversity which was mostly due to a loss of foundational microbiota such as *Lactobacillus* spp. *Veillonella* and *Campylobacter* were also found to be positively associated with erosion, though their role as mediators appears limited. Indeed, a reduction in the abundance of *Lactobacillus* spp. in the porcine antrum and corpus has previously been shown to contribute to gastric ulcer formation in the *pars oesophagea* [34]. A decrease in the abundance of *Lactobacillus* strains is known to attenuate the natural resistance against infection or colonization by pathogens. This is due to a decreased competition for nutrients and epithelial binding sites, less production of antimicrobial factors such as lactic acid and bacteriocins and an increased pH in the gastrointestinal tract, which might create a less hostile environment for pathogens [34, 35]. Members of the genus *Veillonella* are known to naturally inhabit the healthy porcine stomach [36]. These are Gram-negative anaerobic cocci with lactate-fermenting abilities. To our knowledge, *Veillonella* spp. have not yet been associated with gastric ulceration in pigs. Of note, an increased abundance of *Veillonella* spp. has been identified in the gastric microbiome of human *H. pylori*-related gastric cancer patients [37] and has been suggested to be related to changes in the luminal pH [37, 38]. While *Campylobacter* spp. are not normally associated with the porcine gastric microbiome, *C. coli* and *C. jejuni* are known to frequently colonize the porcine intestinal tract [39]. Their association with pathology, such as clinical diarrhea, in pigs is suggested to be low [40]. However, *Campylobacter* infections are one of the known causes of gastroenteritis in human patients [41]. The respective role that each of these species might have in the development of porcine gastric ulceration remains to be explored.

These findings support the importance of finding a way to control *H. suis* infection in pigs. Control strategies should include alternative, non-antibiotic, treatment and vaccination strategies [42, 43]. Preventive antibiotic medication in pigs for *H. suis* eradication is contraindicated taking into account the prudent use of antibiotics in order to avoid antimicrobial resistance in pathogens and the host microbiota [44, 45]. Given the finding that the effect of *H. suis* on porcine gastric ulceration is mediated through changes in the diversity and composition of the pars oesophageal microbiome, administration of certain probiotics may be a potential control strategy. It has already been suggested that the beneficial properties of *Lactobacillus* strains that are abundantly present in healthy porcine stomachs should be looked into [34]. In mice, administration of *L. gasseri* SBT2055 has been shown to significantly reduce gastric colonization with *H. suis* (strain TKY isolated from a cynomolgus monkey) and *H. pylori* (strain SS1) after infection [46]. Moreover, dietary supplementation with this *L. gasseri* strain provided protection against the formation of lymphoid follicles in the gastric mucosa three months after infection with *H. suis* as well as round protrusive lesions in the gastric fundus 12 months after infection, and resulted in significantly better average body weights compared to unsupplemented mice.

The results of the current study confirm the presence of *H. suis* in both the pyloric and fundic gland zones in pigs at slaughter age. Therefore, the hypothesis that *H. suis* colonizes the pyloric gland zone initially and migration to the fundic gland zone takes place at a later age [6] is corroborated by our data. According to the causal inference results, the presence of *H. suis* in the pyloric gland zone in particular seems to impact gastric ulceration and changes in the pars oesophageal microbiome more significantly compared to the presence of *H. suis* in the fundic gland zone.

To the best of our knowledge, this is the first time that absolute numbers of *F. gastrosuis* in the *pars oesophagea* have been reported and associated with the severity of gastric ulceration in the *pars oesophagea*. Earlier, it was reported that *F. gastrosuis* was highly abundant in the gastric microbial community of pooled samples from the *pars oesophagea*, cardiac, fundic and pyloric gland zones of *H. suis*-infected pigs [47]. In another study, it was found that the relative abundance and the colonization rate of *F. gastrosuis* in the *pars oesophagea* in *H. suis*-infected 6- to 8-month-old pigs was higher compared to non-infected pigs. However, in adult sows a lower colonization rate of *F. gastrosuis* in the *pars oesophagea* in *H. suis*-infected pigs was observed compared to non-infected sows. Also, pars oesophageal lesions were more severe in adult sows than in 6- to 8-month-old pigs. It was hypothesized that the colonization rate of *F. gastrosuis* relates to the gastric acid secretion which may be down- or upregulated depending on the duration of *H. suis* infection [7, 8]. Based on this hypothesis, it is possible that the pigs included in this study were already in a later, chronic phase of infection at slaughter age, which may have been accompanied by an upregulation of gastric acid secretion and a decrease in the colonization of *F. gastrosuis* in the *pars oesophagea*. This is consistent with the fact that all pig stomachs included were affected and a high rate of severe lesions in the *pars oesophagea* was observed. The apparent contradiction of the current study stating that more severe gastric lesions in the *pars oesophagea* may be significantly associated with a lower infectious burden of *F. gastrosuis* might be explained by the above hypothesis.

In this study, one biopsy sample of the fundic gland zone was positive in the *ureAB*-based *H. pylori*-specific PCR assay, while negative in the *glmM*-based *H. pylori*-specific PCR assay, indicating that this may concern the presence of *H. pylori*-like organisms, which is in accordance with the results obtained by Cortez Nunes et al*.* [11]. Further evidence concerning the existence of these potentially novel gastric *Helicobacter* species and their possible significance for gastric disease in pigs should be obtained through isolation and genomic characterization of the organisms, ideally in a large multi-farm study.

A strength of the study design is that, besides the type of feed administered, other possibly confounding factors that could cause variation between barns, were controlled for, therefore easing the interpretation of the obtained results. However, other unknown factors which were not explored in the current study might play a role in gastric ulceration of the *pars oesophagea* since this is a pathology of multifactorial origin. Ideally, an in vivo study in pigs experimentally co-infected with *H. suis* and *F. gastrosuis* and divided into two groups fed the two types of feed administered in this study, keeping all other management strategies equal, should be performed. In addition, regular pH measurements during the different phases (acute/chronic) of infection to monitor gastric acid secretion, and identification of the pars oesophageal microbiome composition could be performed to further investigate the hypotheses put forward in this study and earlier studies.

In conclusion, the development of gastric lesions in the *pars oesophagea* indeed has a multifactorial etiology with an important microbial component. The current results hypothesize that *H. suis* infections have a direct causal effect in the development of gastric ulceration, which is mediated by shifts in the pars oesophageal microbiome through alterations in gastric acid secretion. In addition, a pelleted feed may also promote ulceration. Gaining knowledge on how to control *H. suis* infection, maintaining a healthy porcine gastric microbiome and improving feed management of pigs may positively impact pig health.

### Supplementary Information

**Additional file 1. Feed compositions according to the feed labels**.

**Additional file 2. Differentially abundant Amplicon Sequence Variants (ASVs) associated with meal feed and macroscopic lesion score.**

**Additional file 3. Sensitivity analysis for the effect of any unobserved confounders considering the magnitude of the effect of the barns on the pars oesophageal diversity.**

## Data Availability

The datasets used and/or analyzed during the current study are available from the corresponding author upon reasonable request. All of the scripts concerning mediation and differential abundance analyses are also available on GitHub and can be made public for review and publication.
