# Supplementary material for: The role of Helicobacter suis, Fusobacterium gastrosuis, and the pars oesophageal microbiota in gastric ulceration in slaughter pigs receiving meal or pelleted feed
Source: Vet Res. 2024 Feb 5;55:15. doi: 10.1186/s13567-024-01274-1 (PMC10845778; doi:10.1186/s13567-024-01274-1)
Supplement: Supplementary file 1 — Additional file 1. Feed compositions according to the feed labels. [file 13567_2024_1274_MOESM1_ESM.pdf]

Table S1. Feed compositions according to the feed labels

	Meal feed			Pelleted feed		
**Animal weight**	**20-45 kg**	**45-80 kg**	**80-115 kg**	**20-45 kg**	**45-80 kg**	**80-115 kg**
**Analytical safeguards as mentioned on the feed labels (%)**						
Crude protein	16.04	15.00	15.06	16.02	15.00	14.75
Crude fat	4.00	3.85	3.40	3.52	3.30	3.00
Crude ash	5.20	4.72	4.74	5.16	5.36	5.39
Crude fibre	3.76	4.17	4.06	3.80	4.00	4.02
Calcium	0.83	0.72	0.72	0.82	0.72	0.72
Phosphorus	0.38	0.38	0.39	0.38	0.37	0.39
Sodium	0.24	0.25	0.23	0.22	0.25	0.20
Lysine	1.07	0.98	0.88	1.07	0.98	0.87
Methionine	0.37	0.32	0.28	0.37	0.32	0.28
**Ingredient composition as mentioned on the feed labels**						
Wheat	x	x	x	x	x	x
Barley	x	x	x	x	x	x
Toasted soybean meal	x	x	x	x	x	x
Wheat gluten	x	x	x	x	x	x
Cookie meal	x	x	x	x	x	
Corn meal			x			x
Corn	x	x	x	x	x	x
Toasted soybeans	x	x	x	x	x	x
Limestone	x	x	x	x	x	x
Palm oil	x	x	x	x	x	x
Rapeseed meal		x	x		x	x
Molasses	x	x	x	x	x	x
Dried sugar beet pulp	x	x		x		
Sunflower seed meal		x		x		
Wheat bran			x			x
Sodium chloride	x	x	x	x	x	x
Monocalcium phosphate	x			x		
Sodium bicarbonate	x	x	x	x	x	x

x = present according to feed label, empty = absent according to feed label
