# Supplementary figures and images for: The role of Helicobacter suis, Fusobacterium gastrosuis, and the pars oesophageal microbiota in gastric ulceration in slaughter pigs receiving meal or pelleted feed
Source: Vet Res. 2024 Feb 5;55:15. doi: 10.1186/s13567-024-01274-1 (PMC10845778; doi:10.1186/s13567-024-01274-1)

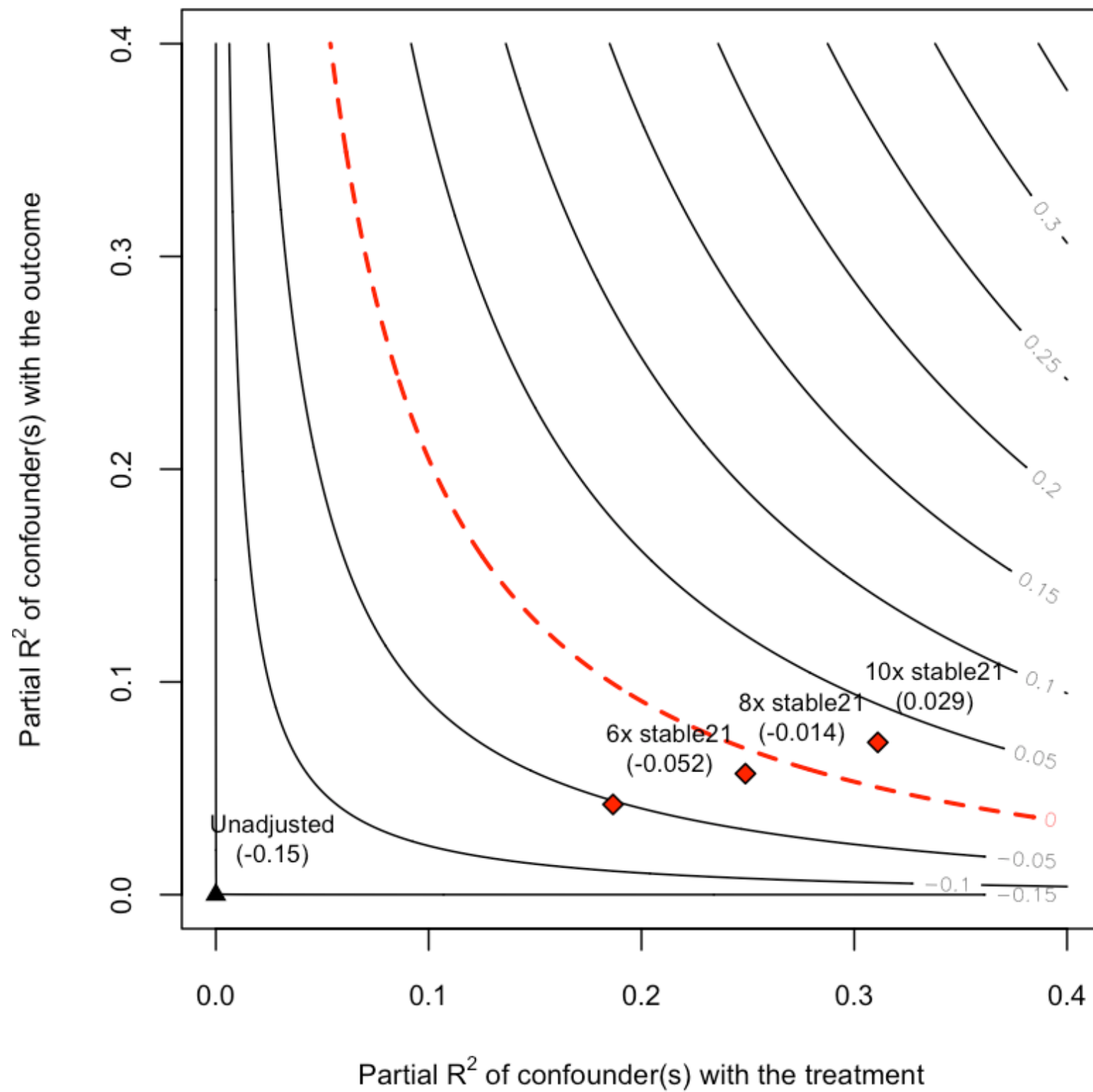

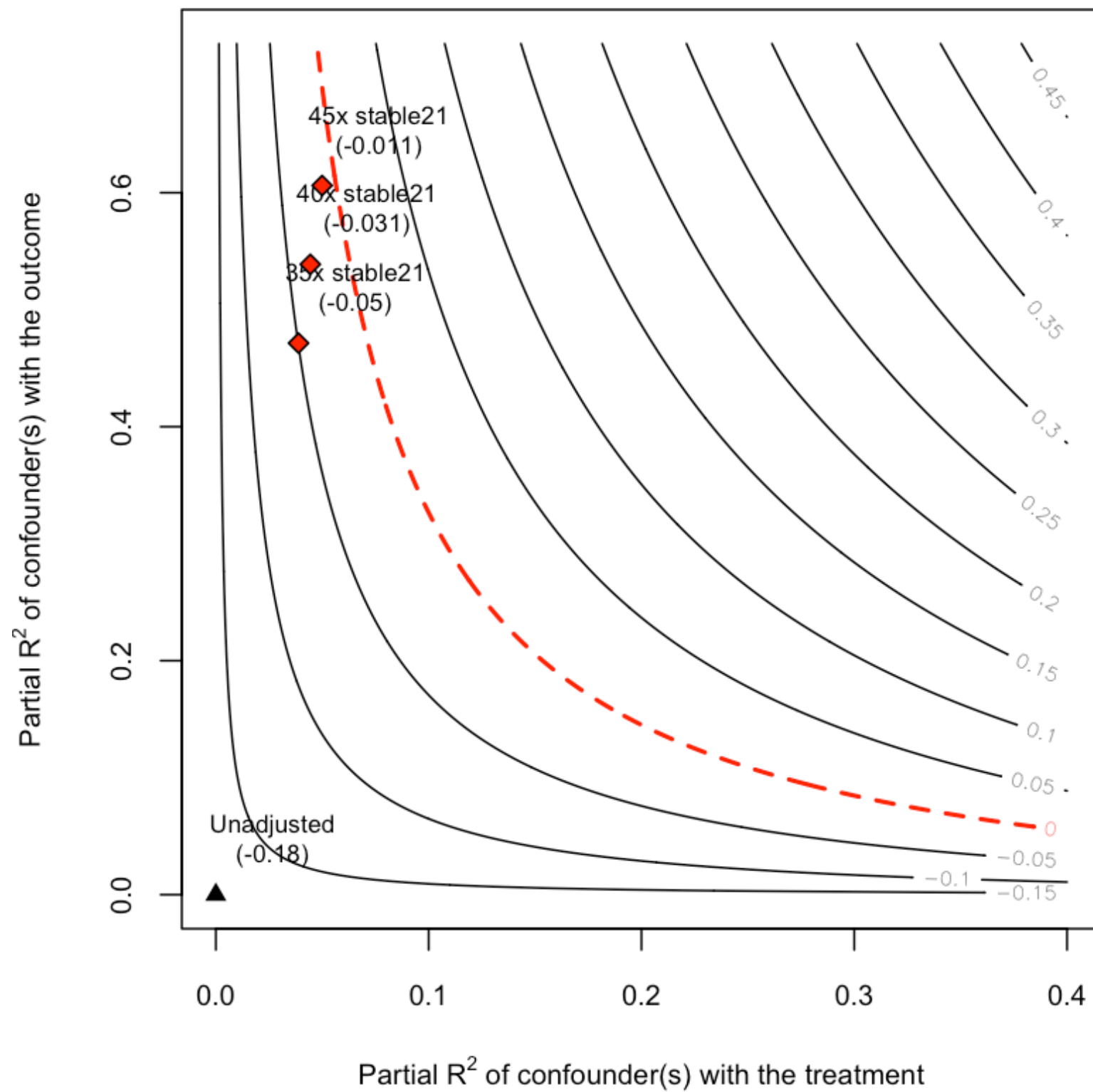

Supplement: Supplementary file 3 — Additional file 3. Sensitivity analysis for the effect of any unobserved confounders considering the magnitude of the effect of the barns on the pars oesophageal diversity. [file 13567_2024_1274_MOESM3_ESM.pdf]
